# Monitoring of Cerebral Blood Flow and Metabolism Bedside in Patients with Subarachnoid Hemorrhage – A Xenon-CT and Microdialysis Study

**DOI:** 10.3389/fneur.2014.00089

**Published:** 2014-06-02

**Authors:** Elham Rostami, Henrik Engquist, Ulf Johnson, Timothy Howells, Elisabeth Ronne-Engström, Pelle Nilsson, Lars Hillered, Anders Lewén, Per Enblad

**Affiliations:** ^1^Section of Neurosurgery, Department of Neuroscience, Uppsala University, Uppsala, Sweden; ^2^Anesthesiology and Intensive Care, Department of Surgical Sciences, Uppsala University, Uppsala, Sweden

**Keywords:** cerebral blood flow, subarachnoid hemorrhage, neurointensive care, Xenon-CT, imaging, vasospasm, microdialysis

## Abstract

Cerebral ischemia is the leading cause of morbidity and mortality following aneurysmal subarachnoid hemorrhage (SAH). Although 70% of the patients show angiographic vasospasm only 30% develop symptomatic vasospasm defined as delayed cerebral ischemia (DCI). Early detection and management of reversible ischemia is of critical importance in patients with SAH. Using a bedside Xenon enhanced computerized tomography (Xenon-CT) scanner makes it possible to measure quantitative regional Cerebral blood flow (CBF) bedside in the neurointensive care setting and intracerebral microdialysis (MD) is a method that offers the possibility to monitor the metabolic state of the brain continuously. Here, we present results from nine SAH patients with both MD monitoring and bedside Xenon-CT measurements. CBF measurements were performed within the first 72 h following bleeding. Six out of nine patients developed DCI at a later stage. Five out of six patients who developed DCI had initial global CBF below 26 ml/100 g/min whereas one had 53 ml/100 g/min. The three patients who did not develop clinical vasospasm all had initial global CBF above 27 ml/100 g/min. High lactate/pyruvate (L/P) ratio was associated with lower CBF values in the area surrounding the catheter. Five out of nine patients had L/P ratio ≥25 and four of these patients had CBF ≤ 22 ml/100 g/min. These preliminary results suggest that patients with initially low global CBF on Xenon-CT may be more likely to develop DCI. Initially low global CBF was accompanied with metabolic disturbances determined by the MD. Most importantly, pathological findings on the Xenon-CT and MD could be observed before any clinical signs of DCI. Combining bedside Xenon-CT and MD was found to be useful and feasible. Further studies are needed to evaluate if DCI can be detected before any other signs of DCI to prevent progress to infarction.

## Introduction

Positron emission tomography (PET) studies have shown that subarachnoid hemorrhage (SAH) causes both global and focal hemodynamic and metabolic disturbances in the brain early after aneurysm rupture (1), which makes the brain vulnerability for secondary insults (2–4). Secondary ischemic brain injury may be caused by cerebral vasospasm and also by various other secondary insults, e.g., high intracranial pressure (ICP), hypotension, hypoxemia, and seizures. Microcirculatory dysfunction, microthrombosis, cortical spreading depression, inflammation, and disturbances in cerebral autoregulation are today considered to be important mechanisms for the development of secondary ischemia in SAH (3, 5). Vasospasm is defined as the narrowing of intracranial arteries. The clinical definition of vasospasm has often been based on the methods applied for diagnosis such as angiography, transcranial Doppler (TCD), symptomatology, and imaging (5). Using clinical definitions, cerebral vasospasm has shown to be the leading cause of morbidity and mortality following aneurysmal SAH (6). It is estimated that 70% of the patients develop vasospasm diagnosed by angiography (7, 8), while only 30–50% develop symptomatic vasospasm (9, 10). This discrepancy between angiographic findings and development of clinical signs is one of the reasons why the role of macrovascular narrowing has been questioned as the major mechanism for the development of ischemia. Current guidelines of the critical care management of patients with SAH recommend the use of the term delayed cerebral ischemia (DCI), defined as focal neurological impairment or decrease of Glasgow coma score, and/or radiological signs of ischemia/infarction (11).

Reduced cerebral blood flow (CBF) becomes symptomatic below the critical threshold for electrical/functional disturbances (12, 13). If sustained, it will lead to irreversible ischemia and finally infarction. Thus early detection of low CBF is of critical importance in patients with SAH.

Currently, the detection of vasospasm is routinely based on the repeated neurological examinations, TCD blood flow velocity measurements and angiography. When treating patients with severe SAH, it would be desirable to have more accurate methods for the measurements of regional and global CBF at the bedside in the neurointensive care (NIC) unit. A few centers have used a bedside Xenon enhanced computerized tomography (Xenon-CT) in the NIC setting, and their experiences indicate that this may be an attractive solution (14–16). Xenon-CT has been shown to be feasible and valuable in detecting early signs of vasospasm/low CBF and to predict development of infarction (17, 18). Hypoperfusion measured by Xenon-CT during the first 24 h following SAH has been shown to correlate with worse outcome (19). We have introduced a new protocol for the management of SAH patients at our department adding bedside Xenon-CT to current neuromonitoring in order to measure CBF. Here, we present our first results from nine patients suffering from severe SAH who were examined with Xenon-CT and simultaneously monitored by intracerebral microdialysis (MD).

## Materials and Methods

### Study population and study design

Nine patients with aneurysmal SAH who were admitted to the Department of Neurosurgery, Uppsala University Hospital, between October 2012 and July 2013 were included in the study (Table 1). All but one of the nine patients were female. The average age was 56.2 ± 13.2.

**Table 1:** **Patient demographics and clinical characteristics of nine SAH patients**.

Patient	Sex	Age	DCI	H&H	CT- Fisher	Treatment	GCS-M in	GCS-M out
1	F	51	No	1	3	Clipping	6	6
2	F	73	Yes	3	4c	Coiling	6	4
3	F	64	Yes	3	4c	Coiling	6	6
4	F	28	No	5	4a	Coiling	1	6
5	F	56	Yes	4	4a	Coiling	5	6
6	F	67	No	2	4a	Coiling	6	4
7	M	54	Yes	4	4a	Coiling	4	6
8	F	64	Yes	1	4a	Coiling	6	5
9	F	49	Yes	2	4a	Coiling	6	6

*The Hunt & Hess grade is based on the status at admission and CT-fishers grade is based on the initial CT. GCS-M, Glasgow coma scale motor response*.

Inclusion criteria were mechanically ventilated SAH patients with intraventricular catheter and MD monitoring who underwent Xenon-CT within 72 h of symptom onset. Patients with a pre-existing neurological deficit or a SAH resulting from trauma or arterio-venous malformation (AVM) were excluded. The SAH was verified by CT scanning and the aneurysm was visualized by a CT angiography or digital subtraction angiography. All patients included in the study were enrolled within 24 h from the onset of SAH. All patients underwent surgical clipping (*n* = 1) or endovascular coiling (*n* = 8) of the ruptured aneurysm within the first day except one patient who underwent endovascular coiling after 2 days.

### Neurointensive care

All SAH patients were managed according to a standardized protocol based on intensive physiological monitoring and aggressive therapy of any derangement to avoid or minimize secondary brain injury (4). All unconscious patients and patients with clinical and radiological signs of intracranial hypertension received a ventriculostomy. If ICP was above 20 mmHg, the drainage system was opened and cerebrospinal fluid drained against a pressure level of 15 mmHg. Hypotension was treated first with albumin 20% and crystalloid solutions and with Dobutamine if needed. The goal was to keep CPP above 60 mmHg. Identified aneurysms were treated early by endovascular coiling or surgical clipping. All patients received nimodipine (Nimotop^®^, Bayer AB, Solna, Sweden). The patients were diagnosed to have DCI when delayed ischemic neurological deterioration/deficits occurred that could not be explained by other reasons, i.e., clot, hydrocephalus, or infection. DCI was treated with hypertensive, hypervolemic, and hemodilution therapy (triple-H therapy) by the administration of Dextran 40 solutions 500 ml/day (Rheomacrodex^®^, 100 mg/ml, Meda AB, Solna, Sweden) and Albumin 100 ml,2× (200 mg/ml, Baxter Medical AB, Kista, Sweden).

### CBF measurements

As a part of the new protocol at our department, Xenon-CT is performed bedside in the NIC unit on all patients with SAH within the first 72 h after admission. CBF measurements using Xenon-CT were performed bedside in our NIC unit according to the principles described by Yonas et al. (20–23). The Xenon gas is a radio opaque, highly lipid soluble, and capable of crossing the BBB. The Kety–Schmidt equation is applied to measure regional and global CBF (24). Stable Xenon at a concentration of 28% was administered to the patients breathing circuit for about 4 min using the Enhancer 3000 and computer software (Diversified Diagnostic Products Inc., Houston, USA). During the Xenon inhalation, CT scans were obtained by the CereTom^®^ (Neurologica, Boston, USA). The Xenon delivery and the CT scans were synchronized by computer software and the resulting radiologic tissue enhancement of the Xenon wash-in enabled CBF (ml/100 g/min) to be calculated and plotted as colored maps in scans at four different levels of the brain (eight scans per level, two baselines and six enhanced, with 10-mm spacing).

**Figure 1:**
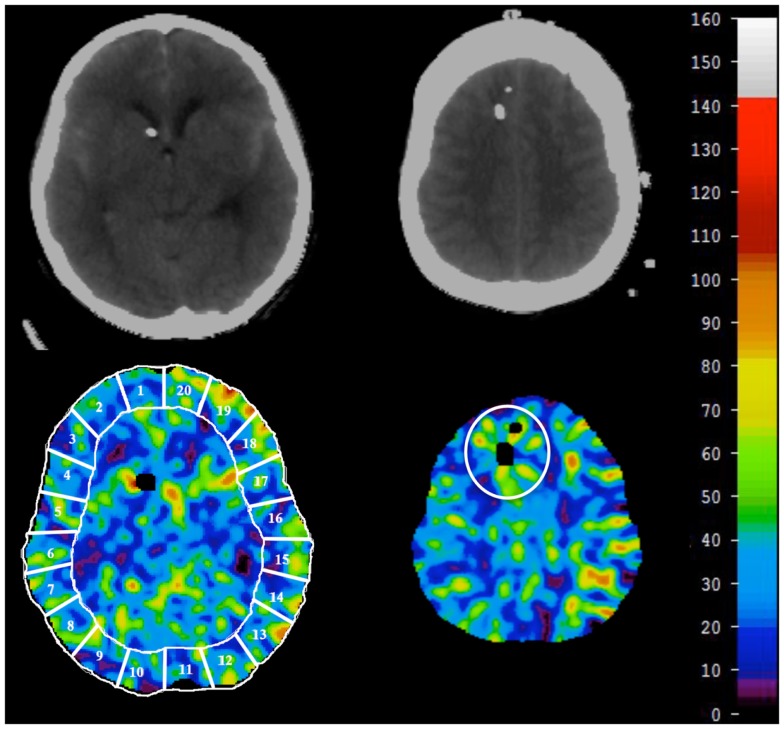
**At each level, CBF in 20 ROIs was calculated and averaged for global CBF (lower left)**. The vascular territories were analyzed as follows: anterior cerebral artery ROI 1–2 (lower right) and 19–20 (lower left), middle cerebral artery ROI 3–8 (lower right) and 13–18 (lower left), and posterior cerebral artery ROI 9–10 (lower right) and 11–12 (lower left). For CBF around the microdialysis catheter, a ROI was manually drawn around the microdialysis catheter after localization on CT-scan (lower right).

Mean blood flow in each of 20 evenly distributed cortical regions (ROIs) was calculated for each level (Figure 1). The global CBF is given as a mean of all four levels. The tip of the MD catheter was identified on the structural CT scans and a ROI was drawn manually for the corresponding area around the MD catheter on the CBF scans (Figure 1). The vascular territories were analyzed as following: anterior cerebral artery ROI 1–2 (right) and 19–20 (left), middle cerebral artery ROI 3–8 (right) and 13–18 (left), and posterior cerebral artery ROI 9–10 (right) and 11–12 (left). Territories with CT-defined hematoma or artifact were noted and excluded.

### Cerebral microdialysis

The cerebral MD technique in NIC has previously been extensively used and described (25, 26). The intracerebral MD catheter was placed in the right frontal lobe cortex through a separate burr hole, anterior to the ventricular drain. The anatomical location of the MD catheters was evaluated by CT scans. For intracerebral monitoring, we used 70 Brain Microdialysis Catheter (M Dialysis AB, Solna, Sweden) with a membrane length of 10 mm and a membrane cutoff of 20,000 Da was used for intracerebral MD. The catheters were perfused with artificial CSF (Na^+^, 147 mM; Ca^2+^, 1.2 mM; Mg^2+^, 0.85 mM; K^+^, 2.7 mM; and Cl^−^, 152 mM). The perfusion rate was 0.3 μl/min using a microinjection pump (CMA-106, M Dialysis AB, Solna, Sweden). MD urea was monitored to validate catheter performance (27). The MD samples were collected on an hourly basis. MD samples for the 3-h period before and 3-h period after the Xenon-CT examination were used for analysis. Interstitial glucose, lactate, pyruvate, glutamate, glycerol, and urea were analyzed enzymatically using a CMA 600 analyzer (M Dialysis AB, Solna, Sweden). Quality control measurements using control samples for the CMA 600 microdialysis analyzer (M Dialysis AB, Solna, Sweden) were run daily.

### Ethics

The Uppsala University Regional Ethical Review Board for clinical research granted permission to undertake the study. Written informed consent was obtained from all patients or their proxy for study participation. The study was also approved by the local Radiation Safety Authority.

## Results

**Figure 2:**
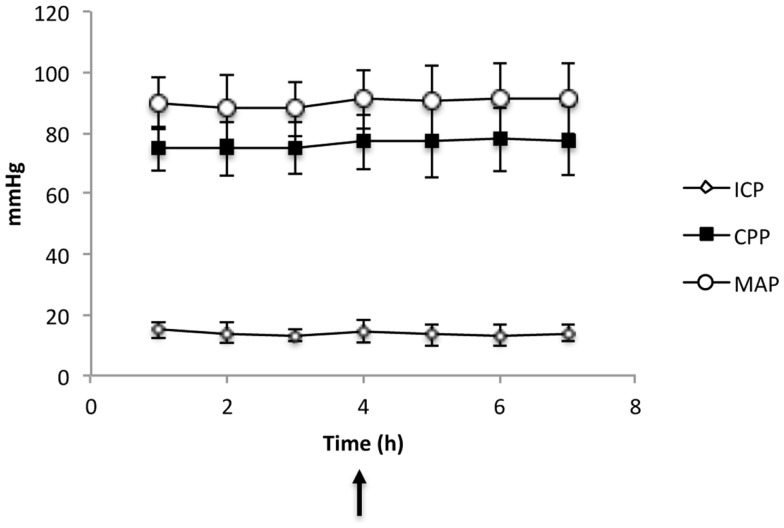
**Physiological parameters (CPP, cerebral perfusion pressure; MAP, mean arterial pressure) and ICP (Intracranial pressure) in all nine patients before and after Xenon-CT (indicated by the arrow)**. The values are given as mean ± SD.

Demographics and clinical characteristics of the nine SAH patients studied are presented in Table 1. The Xenon-CT CBF measurements were performed within the first 3 days (mean 2.9 ± 0.9) after bleeding and no complications were observed during the measurements. The physiological values during the CBF measurements are presented in Table 2. The ICP and CPP were within normal range 13.6 ± 3.4 mmHg and 77 ± 10.2 mmHg, respectively and did not change compared to before and after CBF measurements (Figure 2). None of the patients were hyperventilated during the CBF measurements. The mean pCO_2_ was 5.1 ± 0.3 kPa and mean pO_2_ was 13.9 ± 2.3 kPa. No significant changes could be observed in ICP, CPP, and MAP during and after CBF measurements compared with before. Six out of the nine patients developed DCI according to the applied definition (Table 1).

**Table 2:** **Physiological parameters and neurological grade during the Xenon-CT CBF measurements are presented for each patient**.

Patient	ICP (mmHg)	MAP (mmHg)	CPP (mmHg)	FIO_2_ (%)	pCO_2_ (kPa)	pO_2_ (kPa)	Propofol (mg/kg/h)	GCS-M
1	15.8	104.9	89.1	30	5.1	13.6	5.9	5
2	12.4	107.7	94.3	40	5.8	17.2	2.2	6
3	14.7	90.8	76.4	45	4.8	12.9	3.5	4
4	15.3	84.8	71.9	30	5.4	17.4	5.2	6
5	14.6	96.4	82.2	35	5.0	14.1	3.1	4
6	9.2	76.6	62.5	45	5.4	12.3	1.8	4
7	13.5	80.0	70.3	40	5.2	14.2	2.8	6
8	18.9	87.2	68.4	40	5.0	9.9	3.3	5
9	7.7	85.8	78.1	30	4.7	13.9	3.2	6

*ICP, intracranial pressure; MAP, mean arterial pressure; CPP, cerebral perfusion pressure; FIO_2_, fraction of inspired oxygen; GCS-M, Glasgow coma scale motor score*.

### Global CBF

**Figure 3:**
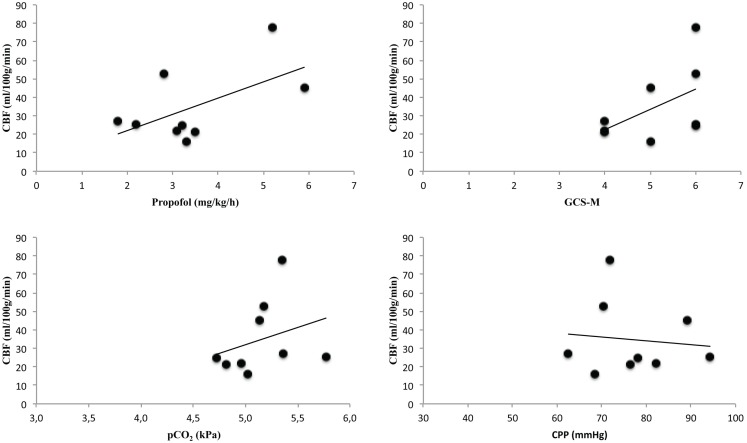
**The figure presents correlations between global cerebral blood flow (CBF) and the amount of sedation (Propofol), neurological grade (GCS-M, Glasgow coma scale motor score), pCO_2_, and cerebral perfusion pressure (CPP) during Xenon-CT measurements**.

**Figure 4:**
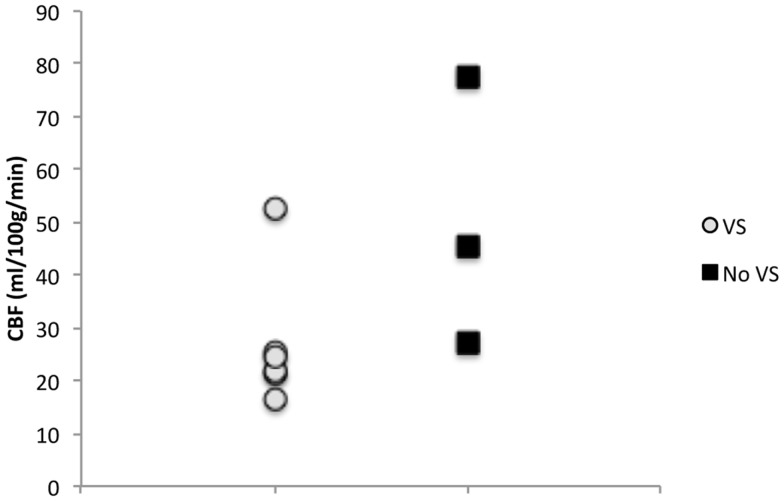
**Six out of nine patients developed DCI while three did not**. Patients that did not develop DCI tended to have higher initial CBF than patients who did develop DCI.

The mean global CBF was 35 ± 20 ml/100 g/min (median = 25, range 64) (Table 3). The correlations between global CBF and neurological grade, CPP, pCO_2_, and amount of sedation, respectively, are shown in Figure 3. Five out of six patients who developed DCI had global CBF below 26 ml/100 g/min whereas one had 53 ml/100 g/min (Figure 4). Those three patients who did not develop DCI all had global CBF above 27 ml/100 g/min. Due to the low number of patients no statistics have been performed.

**Table 3:** **Aneurysm location and CBF in different regions**.

Patient	Aneurysm location	Cerebral blood flow (ml/100 g/min)					
		Ant		Post		Middle		Global	MD
		Right	Left	Right	Left	Right	Left	
1	Left MCA	49 (22)	48 (21)	42 (5)	44 (11)	44 (10)	45 (12)	45.4	34.9
2	ACA	31 (15)	26 (−1)	21 (−20)	23 (−14)	38 (41)	13 (−50)	25.5	23.1
3	ACA	18 (−23)	13 (−44)	23 (−4)	21 (−13)	32 (34)	20 (−16)	21.3	18.5
4	PICA	82 (12)	75 (2)	79 (8)	93 (27)	63 (−14)	74 (1)	77.6	58.6
5	A cerb sup	16 (−24)	24 (15)	27 (26)	23 (9)	20 (−8)	20 (−4)	21.7	19.2
6	ACA	23 (−19)	29 (6)	27 (−4)	27 (−4)	29 (3)	29 (3)	27.2	22.1
7	AcomA	56 (6)	62 (17)	44 (−17)	46 (−12)	53 (0)	54 (2)	52.6	42.6
8	AcomP + ant	15 (4)	14 (−5)	18 (18)	25 (71)	10 (−32)	15 (2)	16.4	10.6
9	ICA right	45 (115)	33 (61)	13 (−40)	15 (−27)	21 (1)	21 (−1)	24.6	24

*The changes in CBF between right and left side in different vascular regions compared to global CBF are presented in bracket (%). R, right; L, left; MCA, middle cerebral artery; AcoA, anterior communicating artery; PcoA, posterior communicating artery; SCA, superior cerebellar artery; PICA, posterior inferior cerebellar artery; MD, microdialysis region*.

### Regional CBF

The results of CBF measurements in different vascular territories for each patient are presented in Table 3. The mean regional CBF was 37 ± 22 (mean ± SD) ml/100 g/min (median = 30, range = 68) in the ACA territory, 34 ± 17 ml/100 g/min (median = 26, range = 80) in the PCA territory, 32 ± 20 ml/100 g/min (median = 29, range = 60) in the MCA, and 28 ± 14 ml/100 g/min (median = 23, range = 48) in the MD region (Table 3). No obvious lateralization of low CBF in relation to the aneurysm location could be seen. There were individual differences in each patient in different vascular territories, e.g., in patient 2 there was a 50% decrease in the left posterior circulation compared to global CBF while there was an increase in the right side. Patient 5 showed a 24% decrease in CBF in the right anterior circulation while there was a 15% increase in the right posterior circulation. Four patients showed >20% difference between the two sides in the anterior circulation, one patient in the posterior circulation and three patients in the middle vascular territory (Table 3).

### Microdialysis and CBF

**Figure 5:**
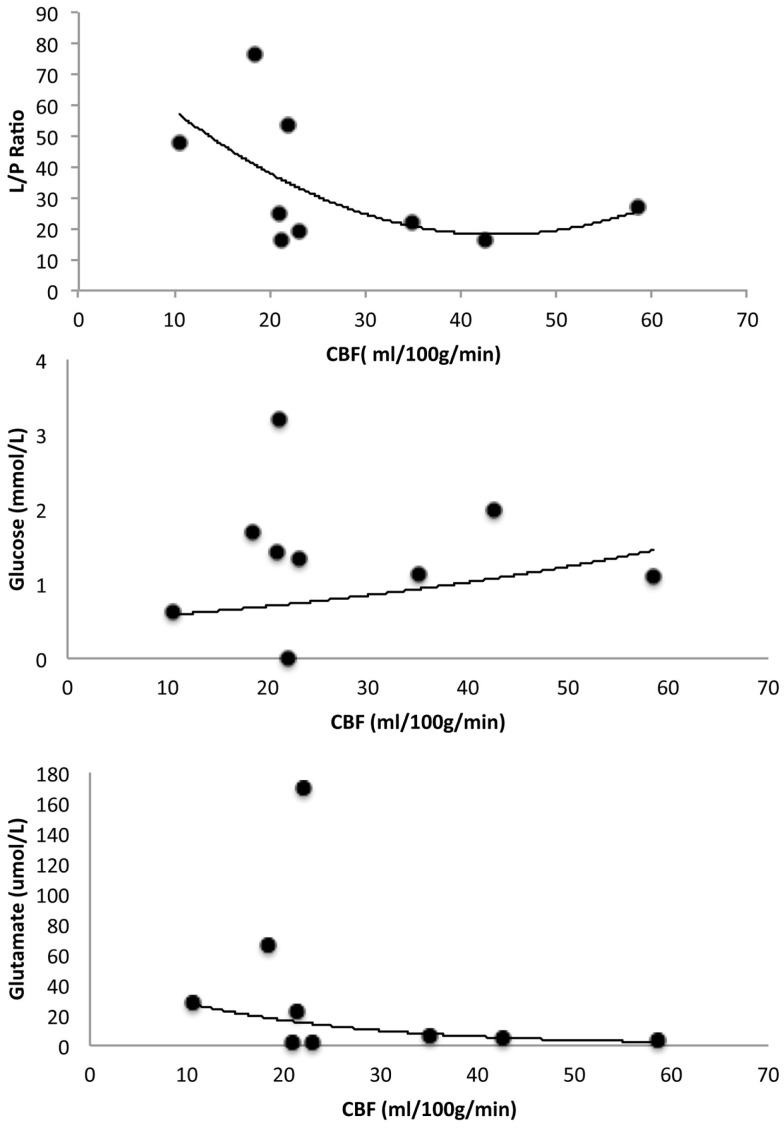
**Illustrates correlation between microdialyzate lactate/pyruvate (L/P) ratio and CBF, glucose and CBF, and glutamate and CBF**. The CBF values represent the area surrounding the microdialysis catheter.

All patients had lower CBF in the region around the catheter (28 ± 14 ml/100 g/min) compared to global CBF (35 ± 20 ml/100 g/min) (Table 3). High lactate/pyruvate (L/P) ratios tended to be correlated with lower CBF values in the area surrounding the catheter and there appeared to be a critical CBF threshold at around 20 ml/100 g/min (Figure 5). Five out of nine patients also had L/P ratio ≥25 four of these patients had CBF ≤ 22 ml/100 g/min. These four patients presented high glutamate levels. No correlation was evident between glucose and regional CBF (Figure 5). In patients 6 and 8, the glucose levels were <1 mmol/l and these patients had CBF ≤ 22 ml/100 g/min, high L/P ratio, and high glutamate levels. In the patients who develop DCI, five had CBF ≤ 23 ml/100 g/min in the region around the catheter. One with CBF 18 ml/100 g/min presented with L/P ratio of 77 and glutamate 66 μmol/l. Patient 8 displayed the lowest CBF in the region around the catheter 10 ml/100 g/min and had a L/P 48 and glutamate 28 μmol/l. Patient 7 showed CBF of 46 ml/100 g/min with low L/P ratio of 16 and glutamate 6 μmol/l.

## Discussion

In this pilot study including nine SAH patients, the first results of Xenon-CT CBF imaging bedside in combination with simultaneous monitoring of cerebral metabolism using MD are presented. We found the use of bedside Xenon-CT feasible and useful in assessing regional CBF in the NIC unit as reported by others (15, 28). No complications occurred during the CBF measurements and the physiological parameters remained within their normal ranges. A previous multicenter study reported the safety of this method with a very low risk of adverse events (14). Using bedside Xenon-CT enables CBF measurements in unstable patients where transportation to the radiology department is associated with risks. The addition of bedside Xenon-CT CBF imaging to multimodality monitoring creates new possibilities for more accurate surveillance and guidance of treatment in NIC. More experience with the technique is needed in order to develop new algorithms for the NIC management.

The serious consequences of DCI following SAH are well known, but it is difficult to identify patients at risk. Our preliminary results suggest that patients with initial low global CBF, below 27 ml/100 g/min, on Xenon-CT may be more likely to develop DCI later. Previous studies using Xenon-CT have shown that cortical CBF in awake normal subjects is 52 ± 10 ml/100 g/min (21). In a study comparing comatose patients following head injury and normal subjects, a CBF threshold of 55.3 ml/100 g/min was defined as hyperemia derived from normal CBF distribution (29). This study suggested CBF values 33.9–55.3 ml/100 g/min as “relative hyperemia”. CBF threshold values vary among different authors. However, studies in animal and humans have shown that CBF < 6 ml/100 g/min indicates severe ischemia and will lead to infarction, CBF >6 and <18 ml/100 g/min are considered as reversible ischemia and CBF >18 and <33.9 ml/100 g/min as reduced flow (29–31). In patients with clinical signs of DCI, low regional CBF levels were measured by Xenon-CT and regions with CBF below 15 ml/100 g/min developed to infarction (18). Reduced global CBF (>18 and <33.9 ml/100 g/min) was seen in patients who developed DCI in the current study when Xenon-CT was performed within the first 72 h. Currently, the triple-H therapy is initiated when clinical signs of vasospasm occur. However, if further studies confirm and demonstrate significant correlation between low initial CBF and development of vasospasm, prophylactic treatment could be initiated at an earlier stage in vulnerable patients at risk of irreversible cerebral damage. Furthermore, the effect of vasospasm therapy could also be evaluated.

The regional analysis of CBF showed decreased CBF in one or more of the vascular territories in most of the SAH patients. This finding is in accordance with an earlier PET study by Frykholm and coll. demonstrating that heterogeneous disturbances of regional CBF were present early after hemorrhage (1). The phenomenon may also be a warning of future risk of vasospasm and/or an indication of an increased vulnerability for various secondary insults (2). The underlying mechanisms for the observed reduced CBF early after SAH deserve further investigation. Interestingly, the suggested mechanisms may be disturbances in microcirculation and occurrence of microthrombosis that leads to reduce blood flow (32–35). This may also explain the findings of reduced CBF in the current study.

Using MD, we observed high L/P ratio and high glutamate levels when regional CBF was below 22 ml/100 g/min in the MD region. Two previous smaller studies have combined MD with Xenon-CT in SAH patients. The first study included 16 SAH patients that underwent Xenon-CT during day 3–10. No correlation could be found between L/P ratio and clinical status (36). Two patients in this study who developed infarction in the region of MD catheter had high glutamate levels. However, this study in contrast to the current did not correlate CBF with L/P ratio and CBF measurements were later than 72 h. In the second study, Al-Rawi et al. investigated the effect of hypertonic saline infusion on substrate delivery and metabolism by using MD and brain tissue oxygen sensor (37). A decrease of L/P ratio was observed in 9 out of 14 SAH patients after hypertonic saline infusion and seven out of nine patients had an increase in CBF according to Xenon-CT. Previous studies have also combined MD with PET in order to assess both CBF and the metabolic state in SAH patients (38–40). Interestingly, the L/P ratio has shown to be the most sensitive and specific MD parameter to indicate ischemia (40). Glutamate has been proposed as an early marker of threatening ischemia in SAH monitored by TCD (41). Using PET and MD glutamate showed high sensitivity for detecting a critically reduced regional CBF below 20 ml/100 g/min (40). These studies are in accordance with findings in animal studies using PET and MD (42).

We did not observe an evident correlation between glucose and CBF, however, two patients with glucose levels <1 mmol/l had CBF ≤ 22 ml/100 g/min, high L/P ratio and high glutamate levels. A low MD glucose level has been reported in SAH patients who develop infarct, and zero values of glucose were detected in patients with unfavorable outcome (43). Low regional brain glucose can depend on low CBF as well as hypo- and hypermetabolism.

## Conclusion

The bedside Xenon-CT can be used safely for SAH patients in conjunction with MD monitoring of cerebral metabolism. The current pilot study includes few patients and therefore the results must be interpreted with caution. The results indicate that reduced CBF during the 72 h following symptom onset may be associated with increased risk of DCI and also with elevated L/P ratio, which is a marker of disturbed energy metabolism.

## Conflict of Interest Statement

The authors declare that the research was conducted in the absence of any commercial or financial relationships that could be construed as a potential conflict of interest.
